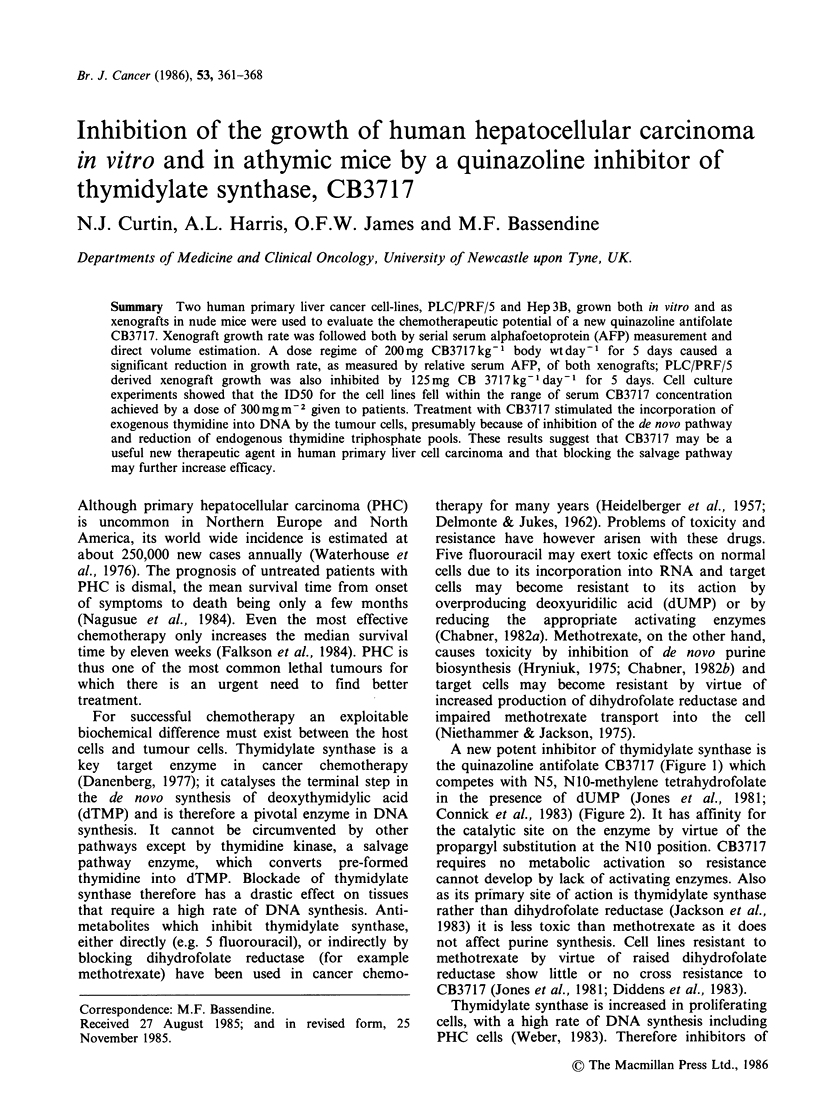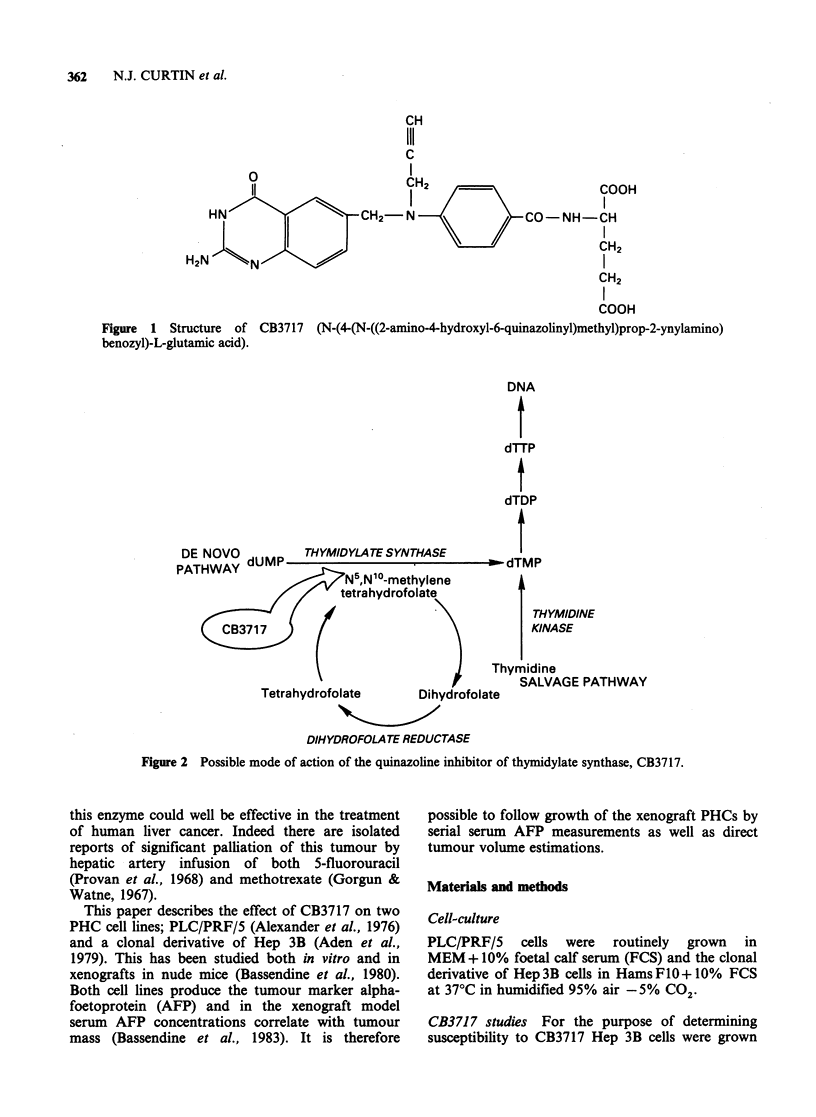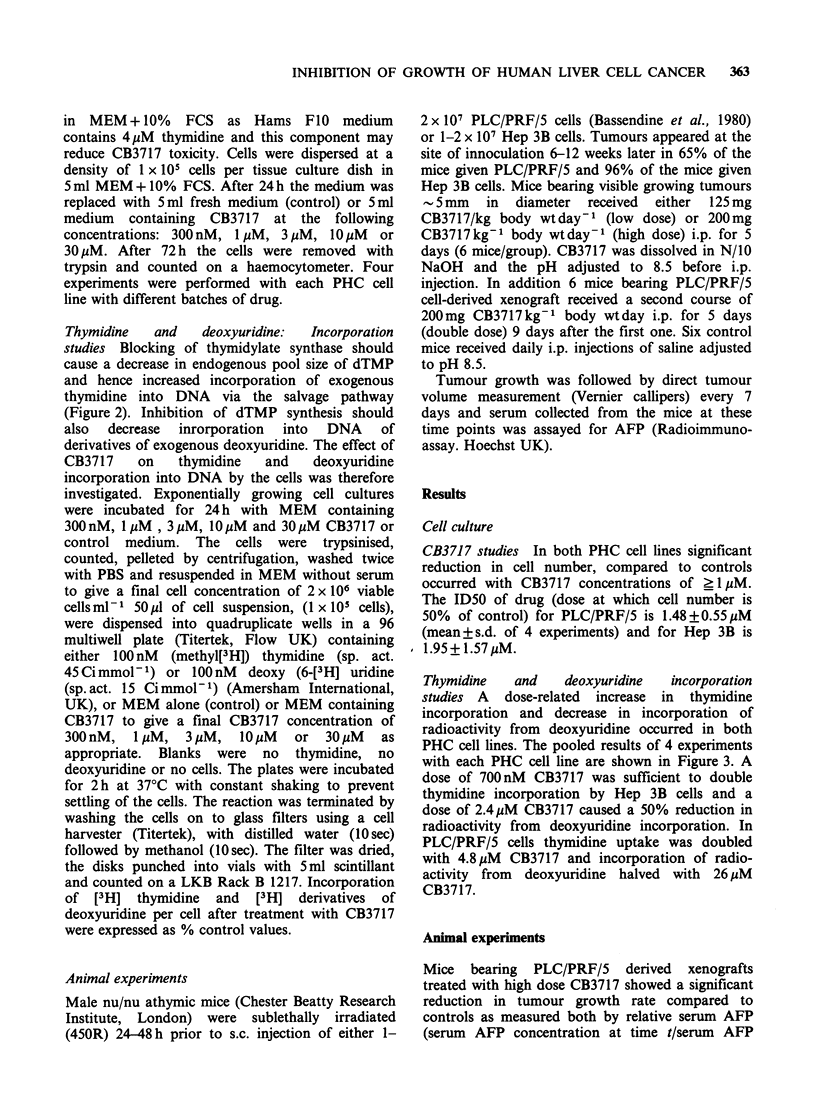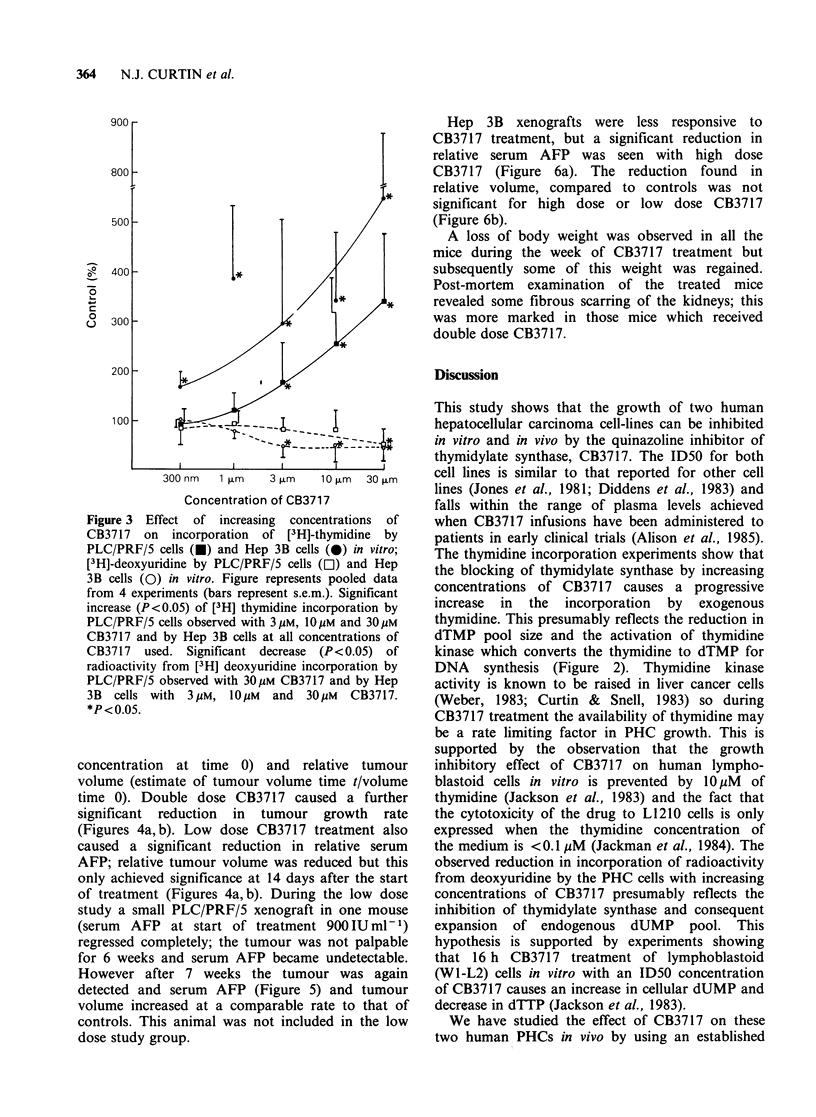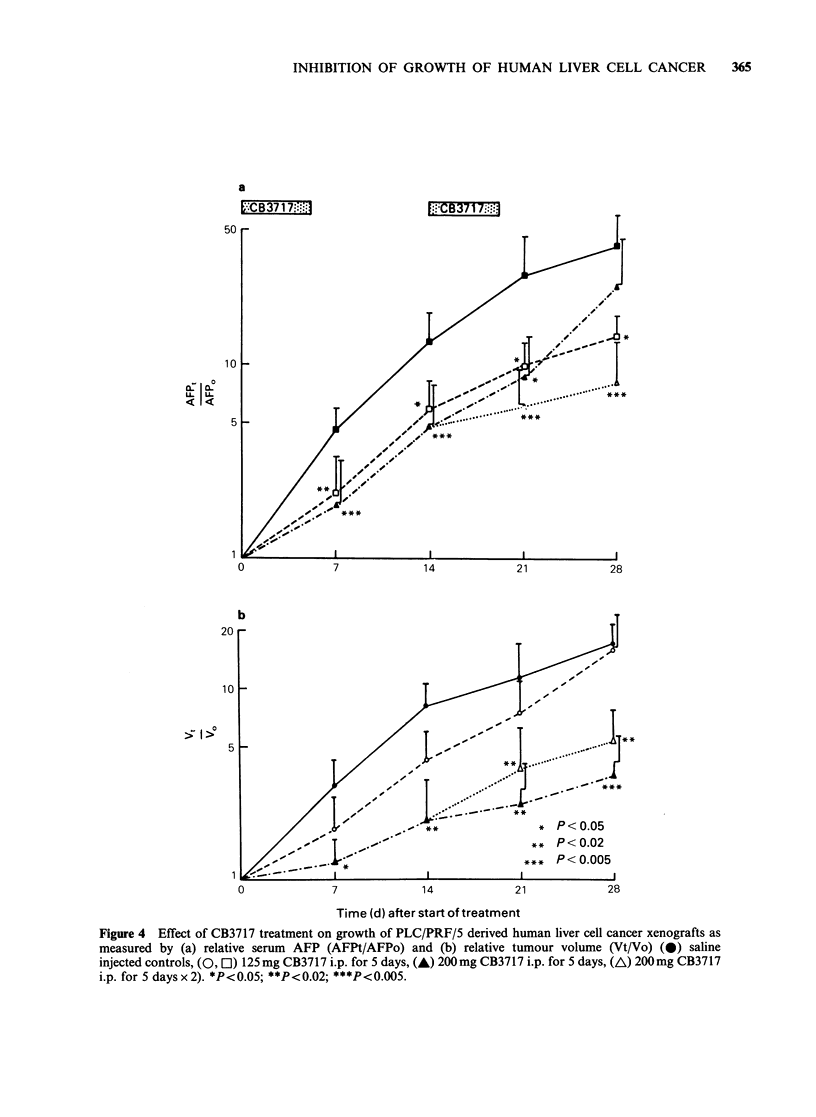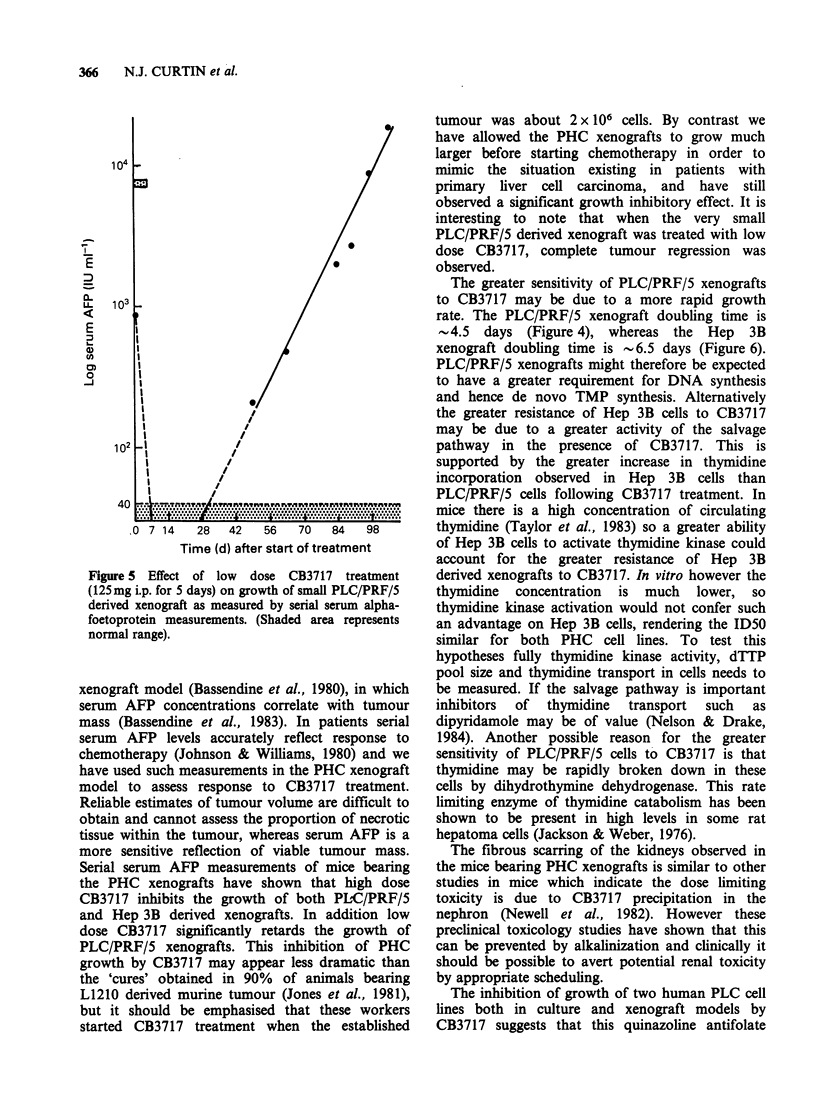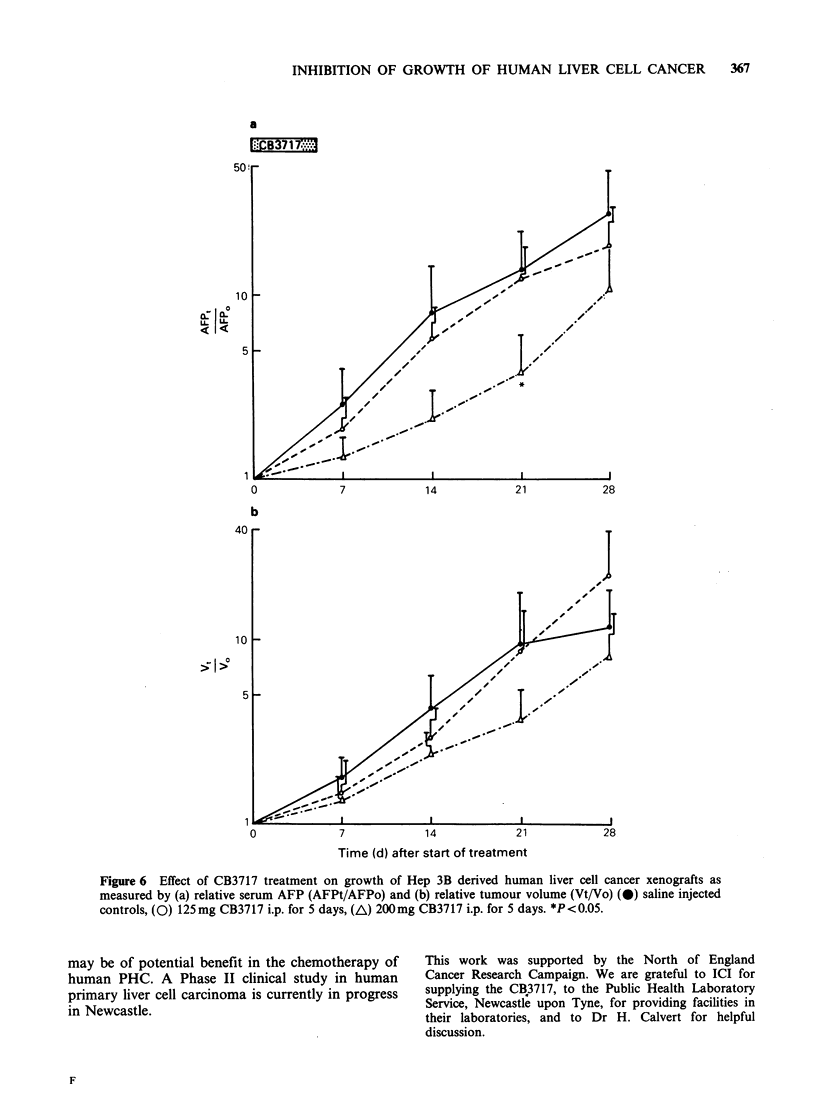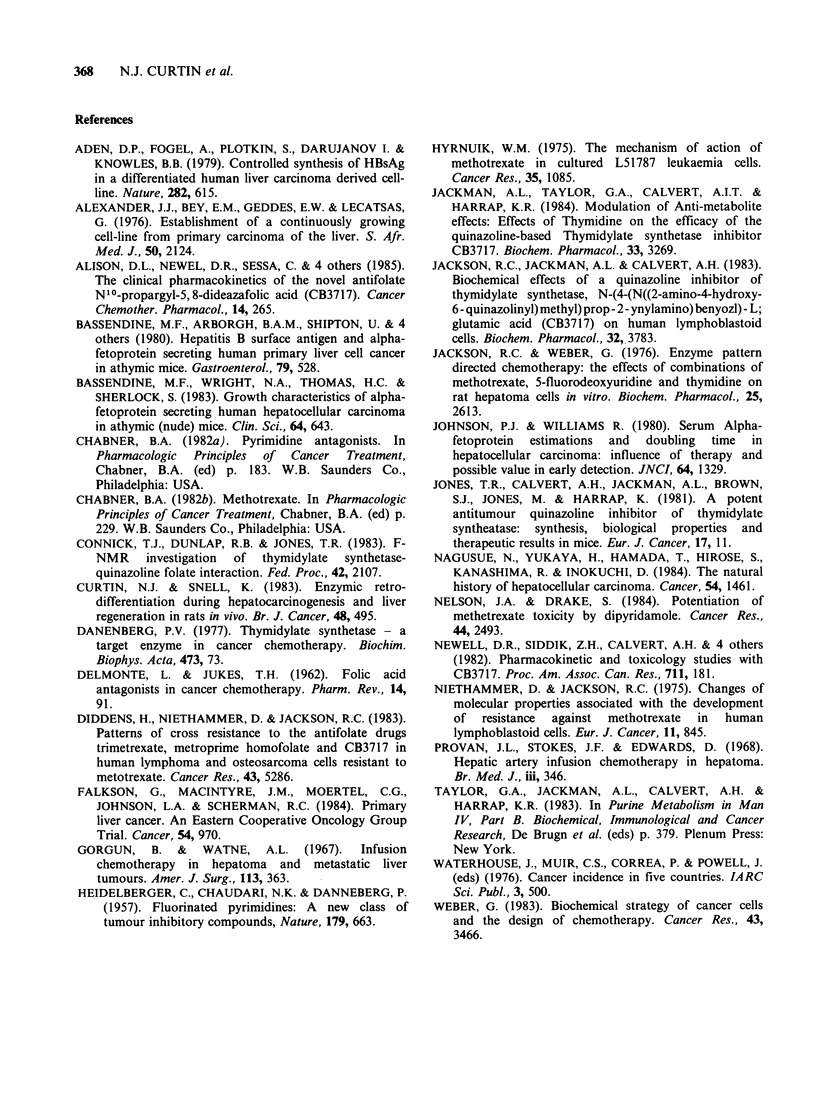# Inhibition of the growth of human hepatocellular carcinoma in vitro and in athymic mice by a quinazoline inhibitor of thymidylate synthase, CB3717.

**DOI:** 10.1038/bjc.1986.60

**Published:** 1986-03

**Authors:** N. J. Curtin, A. L. Harris, O. F. James, M. F. Bassendine

## Abstract

Two human primary liver cancer cell-lines, PLC/PRF/5 and Hep 3B, grown both in vitro and as xenografts in nude mice were used to evaluate the chemotherapeutic potential of a new quinazoline antifolate CB3717. Xenograft growth rate was followed both by serial serum alphafoetoprotein (AFP) measurement and direct volume estimation. A dose regime of 200 mg CB3717 kg-1 body wt day-1 for 5 days caused a significant reduction in growth rate, as measured by relative serum AFP, of both xenografts; PLC/PRF/5 derived xenograft growth was also inhibited by 125 mg CB 3717 kg-1 day-1 for 5 days. Cell culture experiments showed that the ID50 for the cell lines fell within the range of serum CB3717 concentration achieved by a dose of 300 mg m-2 given to patients. Treatment with CB3717 stimulated the incorporation of exogenous thymidine into DNA by the tumour cells, presumably because of inhibition of the de novo pathway and reduction of endogenous thymidine triphosphate pools. These results suggest that CB3717 may be a useful new therapeutic agent in human primary liver cell carcinoma and that blocking the salvage pathway may further increase efficacy.


*Br. J. Cancer* (1986), **53**, 361–368

### Inhibition of the growth of human hepatocellular carcinoma *in vitro* and in athymic mice by a quinazoline inhibitor of thymidylate synthase, CB3717

N.J. Curtin, A.L. Harris, O.F.W. James and M.F. Bassendine

*Departments of Medicine and Clinical Oncology, University of Newcastle upon Tyne, UK.*

**Summary** Two human primary liver cancer cell-lines, PLC/PRF/5 and Hep 3B, grown both *in vitro* and as xenografts in nude mice were used to evaluate the chemotherapeutic potential of a new quinazoline antifolate CB3717. Xenograft growth rate was followed both by serial serum alphafoetoprotein (AFP) measurement and direct volume estimation. A dose regime of 200 mg CB3717 $kg^{-1}$ body wt $day^{-1}$ for 5 days caused a significant reduction in growth rate, as measured by relative serum AFP, of both xenografts; PLC/PRF/5 derived xenograft growth was also inhibited by 125 mg CB 3717 $kg^{-1}$ $day^{-1}$ for 5 days. Cell culture experiments showed that the ID50 for the cell lines fell within the range of serum CB3717 concentration achieved by a dose of 300 mg $m^{-2}$ given to patients. Treatment with CB3717 stimulated the incorporation of exogenous thymidine into DNA by the tumour cells, presumably because of inhibition of the *de novo* pathway and reduction of endogenous thymidine triphosphate pools. These results suggest that CB3717 may be a useful new therapeutic agent in human primary liver cell carcinoma and that blocking the salvage pathway may further increase efficacy.

Although primary hepatocellular carcinoma (PHC) is uncommon in Northern Europe and North America, its world wide incidence is estimated at about 250,000 new cases annually (Waterhouse *et al.*, 1976). The prognosis of untreated patients with PHC is dismal, the mean survival time from onset of symptoms to death being only a few months (Nagusue *et al.*, 1984). Even the most effective chemotherapy only increases the median survival time by eleven weeks (Falkson *et al.*, 1984). PHC is thus one of the most common lethal tumours for which there is an urgent need to find better treatment.

For successful chemotherapy an exploitable biochemical difference must exist between the host cells and tumour cells. Thymidylate synthase is a key target enzyme in cancer chemotherapy (Danenberg, 1977); it catalyses the terminal step in the *de novo* synthesis of deoxythymidylic acid (dTMP) and is therefore a pivotal enzyme in DNA synthesis. It cannot be circumvented by other pathways except by thymidine kinase, a salvage pathway enzyme, which converts pre-formed thymidine into dTMP. Blockade of thymidylate synthase therefore has a drastic effect on tissues that require a high rate of DNA synthesis. Antimetabolites which inhibit thymidylate synthase, either directly (e.g. 5 fluorouracil), or indirectly by blocking dihydrofolate reductase (for example methotrexate) have been used in cancer chemotherapy for many years (Heidelberger *et al.*, 1957; Delmonte & Jukes, 1962). Problems of toxicity and resistance have however arisen with these drugs. Five fluorouracil may exert toxic effects on normal cells due to its incorporation into RNA and target cells may become resistant to its action by overproducing deoxyuridilic acid (dUMP) or by reducing the appropriate activating enzymes (Chabner, 1982*a*). Methotrexate, on the other hand, causes toxicity by inhibition of *de novo* purine biosynthesis (Hryniuk, 1975; Chabner, 1982*b*) and target cells may become resistant by virtue of increased production of dihydrofolate reductase and impaired methotrexate transport into the cell (Niethammer & Jackson, 1975).

A new potent inhibitor of thymidylate synthase is the quinazoline antifolate CB3717 (Figure 1) which competes with N5, N10-methylene tetrahydrofolate in the presence of dUMP (Jones *et al.*, 1981; Connick *et al.*, 1983) (Figure 2). It has affinity for the catalytic site on the enzyme by virtue of the propargyl substitution at the N10 position. CB3717 requires no metabolic activation so resistance cannot develop by lack of activating enzymes. Also as its primary site of action is thymidylate synthase rather than dihydrofolate reductase (Jackson *et al.*, 1983) it is less toxic than methotrexate as it does not affect purine synthesis. Cell lines resistant to methotrexate by virtue of raised dihydrofolate reductase show little or no cross resistance to CB3717 (Jones *et al.*, 1981; Diddens *et al.*, 1983).

Thymidylate synthase is increased in proliferating cells, with a high rate of DNA synthesis including PHC cells (Weber, 1983). Therefore inhibitors of

Correspondence: M.F. Bassendine.
Received 27 August 1985; and in revised form, 25 November 1985.

© The Macmillan Press Ltd., 1986

**Figure 1** Structure of CB3717 (N-(4-(N-((2-amino-4-hydroxyl-6-quinazolinyl)methyl)prop-2-ynylamino) benozyl)-L-glutamic acid).

**Figure 2** Possible mode of action of the quinazoline inhibitor of thymidylate synthase, CB3717.

this enzyme could well be effective in the treatment of human liver cancer. Indeed there are isolated reports of significant palliation of this tumour by hepatic artery infusion of both 5-fluorouracil (Provan *et al.*, 1968) and methotrexate (Gorgun & Watne, 1967).

This paper describes the effect of CB3717 on two PHC cell lines; PLC/PRF/5 (Alexander *et al.*, 1976) and a clonal derivative of Hep 3B (Aden *et al.*, 1979). This has been studied both *in vitro* and in xenografts in nude mice (Bassendine *et al.*, 1980). Both cell lines produce the tumour marker alpha-foetoprotein (AFP) and in the xenograft model serum AFP concentrations correlate with tumour mass (Bassendine *et al.*, 1983). It is therefore possible to follow growth of the xenograft PHCs by serial serum AFP measurements as well as direct tumour volume estimations.

### Materials and methods

### Cell-culture

PLC/PRF/5 cells were routinely grown in MEM + 10% foetal calf serum (FCS) and the clonal derivative of Hep3B cells in Hams F10 + 10% FCS at 37°C in humidified 95% air −5% $CO_2$.

*CB3717 studies* For the purpose of determining susceptibility to CB3717 Hep 3B cells were grown in MEM + 10% FCS as Hams F10 medium contains 4 $\mu$M thymidine and this component may reduce CB3717 toxicity. Cells were dispersed at a density of $1 \times 10^5$ cells per tissue culture dish in 5 ml MEM + 10% FCS. After 24 h the medium was replaced with 5 ml fresh medium (control) or 5 ml medium containing CB3717 at the following concentrations: 300 nM, 1 $\mu$M, 3 $\mu$M, 10 $\mu$M or 30 $\mu$M. After 72 h the cells were removed with trypsin and counted on a haemocytometer. Four experiments were performed with each PHC cell line with different batches of drug.

*Thymidine and deoxyuridine: Incorporation studies* Blocking of thymidylate synthase should cause a decrease in endogenous pool size of dTMP and hence increased incorporation of exogenous thymidine into DNA via the salvage pathway (Figure 2). Inhibition of dTMP synthesis should also decrease inrorporation into DNA of derivatives of exogenous deoxyuridine. The effect of CB3717 on thymidine and deoxyuridine incorporation into DNA by the cells was therefore investigated. Exponentially growing cell cultures were incubated for 24 h with MEM containing 300 nM, 1 $\mu$M, 3 $\mu$M, 10 $\mu$M and 30 $\mu$M CB3717 or control medium. The cells were trypsinised, counted, pelleted by centrifugation, washed twice with PBS and resuspended in MEM without serum to give a final cell concentration of $2 \times 10^6$ viable cells ml$^{-1}$ 50 $\mu$l of cell suspension, ($1 \times 10^5$ cells), were dispensed into quadruplicate wells in a 96 multiwell plate (Titertek, Flow UK) containing either 100 nM (methyl[$^3$H]) thymidine (sp. act. 45 Ci mmol$^{-1}$) or 100 nM deoxy (6-[$^3$H] uridine (sp. act. 15 Ci mmol$^{-1}$) (Amersham International, UK), or MEM alone (control) or MEM containing CB3717 to give a final CB3717 concentration of 300 nM, 1 $\mu$M, 3 $\mu$M, 10 $\mu$M or 30 $\mu$M as appropriate. Blanks were no thymidine, no deoxyuridine or no cells. The plates were incubated for 2 h at 37°C with constant shaking to prevent settling of the cells. The reaction was terminated by washing the cells on to glass filters using a cell harvester (Titertek), with distilled water (10 sec) followed by methanol (10 sec). The filter was dried, the disks punched into vials with 5 ml scintillant and counted on a LKB Rack B 1217. Incorporation of [$^3$H] thymidine and [$^3$H] derivatives of deoxyuridine per cell after treatment with CB3717 were expressed as % control values.

*Animal experiments*

Male nu/nu athymic mice (Chester Beatty Research Institute, London) were sublethally irradiated (450R) 24–48 h prior to s.c. injection of either 1–$2 \times 10^7$ PLC/PRF/5 cells (Bassendine *et al.*, 1980) or 1–$2 \times 10^7$ Hep 3B cells. Tumours appeared at the site of innoculation 6–12 weeks later in 65% of the mice given PLC/PRF/5 and 96% of the mice given Hep 3B cells. Mice bearing visible growing tumours $\sim$5 mm in diameter received either 125 mg CB3717/kg body wt day$^{-1}$ (low dose) or 200 mg CB3717 kg$^{-1}$ body wt day$^{-1}$ (high dose) i.p. for 5 days (6 mice/group). CB3717 was dissolved in N/10 NaOH and the pH adjusted to 8.5 before i.p. injection. In addition 6 mice bearing PLC/PRF/5 cell-derived xenograft received a second course of 200 mg CB3717 kg$^{-1}$ body wt day i.p. for 5 days (double dose) 9 days after the first one. Six control mice received daily i.p. injections of saline adjusted to pH 8.5.

Tumour growth was followed by direct tumour volume measurement (Vernier callipers) every 7 days and serum collected from the mice at these time points was assayed for AFP (Radioimmunoassay. Hoechst UK).

### Results

*Cell culture*

*CB3717 studies* In both PHC cell lines significant reduction in cell number, compared to controls occurred with CB3717 concentrations of $\geq 1$ $\mu$M. The ID50 of drug (dose at which cell number is 50% of control) for PLC/PRF/5 is $1.48 \pm 0.55$ $\mu$M (mean $\pm$ s.d. of 4 experiments) and for Hep 3B is $1.95 \pm 1.57$ $\mu$M.

*Thymidine and deoxyuridine incorporation studies* A dose-related increase in thymidine incorporation and decrease in incorporation of radioactivity from deoxyuridine occurred in both PHC cell lines. The pooled results of 4 experiments with each PHC cell line are shown in Figure 3. A dose of 700 nM CB3717 was sufficient to double thymidine incorporation by Hep 3B cells and a dose of 2.4 $\mu$M CB3717 caused a 50% reduction in radioactivity from deoxyuridine incorporation. In PLC/PRF/5 cells thymidine uptake was doubled with 4.8 $\mu$M CB3717 and incorporation of radioactivity from deoxyuridine halved with 26 $\mu$M CB3717.

### Animal experiments

Mice bearing PLC/PRF/5 derived xenografts treated with high dose CB3717 showed a significant reduction in tumour growth rate compared to controls as measured both by relative serum AFP (serum AFP concentration at time *t*/serum AFP

**Figure 3** Effect of increasing concentrations of CB3717 on incorporation of [$^3$H]-thymidine by PLC/PRF/5 cells (■) and Hep 3B cells (●) *in vitro*; [$^3$H]-deoxyuridine by PLC/PRF/5 cells (□) and Hep 3B cells (○) *in vitro*. Figure represents pooled data from 4 experiments (bars represent s.e.m.). Significant increase ($P<0.05$) of [$^3$H] thymidine incorporation by PLC/PRF/5 cells observed with 3 μM, 10 μM and 30 μM CB3717 and by Hep 3B cells at all concentrations of CB3717 used. Significant decrease ($P<0.05$) of radioactivity from [$^3$H] deoxyuridine incorporation by PLC/PRF/5 observed with 30 μM CB3717 and by Hep 3B cells with 3 μM, 10 μM and 30 μM CB3717. *$P<0.05$.

concentration at time 0) and relative tumour volume (estimate of tumour volume time *t*/volume time 0). Double dose CB3717 caused a further significant reduction in tumour growth rate (Figures 4a, b). Low dose CB3717 treatment also caused a significant reduction in relative serum AFP; relative tumour volume was reduced but this only achieved significance at 14 days after the start of treatment (Figures 4a, b). During the low dose study a small PLC/PRF/5 xenograft in one mouse (serum AFP at start of treatment 900 IU ml$^{-1}$) regressed completely; the tumour was not palpable for 6 weeks and serum AFP became undetectable. However after 7 weeks the tumour was again detected and serum AFP (Figure 5) and tumour volume increased at a comparable rate to that of controls. This animal was not included in the low dose study group.

Hep 3B xenografts were less responsive to CB3717 treatment, but a significant reduction in relative serum AFP was seen with high dose CB3717 (Figure 6a). The reduction found in relative volume, compared to controls was not significant for high dose or low dose CB3717 (Figure 6b).

A loss of body weight was observed in all the mice during the week of CB3717 treatment but subsequently some of this weight was regained. Post-mortem examination of the treated mice revealed some fibrous scarring of the kidneys; this was more marked in those mice which received double dose CB3717.

### Discussion

This study shows that the growth of two human hepatocellular carcinoma cell-lines can be inhibited *in vitro* and *in vivo* by the quinazoline inhibitor of thymidylate synthase, CB3717. The ID50 for both cell lines is similar to that reported for other cell lines (Jones *et al.*, 1981; Diddens *et al.*, 1983) and falls within the range of plasma levels achieved when CB3717 infusions have been administered to patients in early clinical trials (Alison *et al.*, 1985). The thymidine incorporation experiments show that the blocking of thymidylate synthase by increasing concentrations of CB3717 causes a progressive increase in the incorporation by exogenous thymidine. This presumably reflects the reduction in dTMP pool size and the activation of thymidine kinase which converts the thymidine to dTMP for DNA synthesis (Figure 2). Thymidine kinase activity is known to be raised in liver cancer cells (Weber, 1983; Curtin & Snell, 1983) so during CB3717 treatment the availability of thymidine may be a rate limiting factor in PHC growth. This is supported by the observation that the growth inhibitory effect of CB3717 on human lymphoblastoid cells *in vitro* is prevented by 10 μM of thymidine (Jackson *et al.*, 1983) and the fact that the cytotoxicity of the drug to L1210 cells is only expressed when the thymidine concentration of the medium is <0.1 μM (Jackman *et al.*, 1984). The observed reduction in incorporation of radioactivity from deoxyuridine by the PHC cells with increasing concentrations of CB3717 presumably reflects the inhibition of thymidylate synthase and consequent expansion of endogenous dUMP pool. This hypothesis is supported by experiments showing that 16 h CB3717 treatment of lymphoblastoid (W1-L2) cells *in vitro* with an ID50 concentration of CB3717 causes an increase in cellular dUMP and decrease in dTTP (Jackson *et al.*, 1983).

We have studied the effect of CB3717 on these two human PHCs *in vivo* by using an established

**Figure 4** Effect of CB3717 treatment on growth of PLC/PRF/5 derived human liver cell cancer xenografts as measured by (a) relative serum AFP (AFPt/AFPo) and (b) relative tumour volume (Vt/Vo) (●) saline injected controls, (○, □) 125 mg CB3717 i.p. for 5 days, (▲) 200 mg CB3717 i.p. for 5 days, (△) 200 mg CB3717 i.p. for 5 days × 2). $^{*}P<0.05$; $^{**}P<0.02$; $^{***}P<0.005$.

**Figure 5** Effect of low dose CB3717 treatment (125 mg i.p. for 5 days) on growth of small PLC/PRF/5 derived xenograft as measured by serial serum alpha-foetoprotein measurements. (Shaded area represents normal range).

xenograft model (Bassendine *et al.*, 1980), in which serum AFP concentrations correlate with tumour mass (Bassendine *et al.*, 1983). In patients serial serum AFP levels accurately reflect response to chemotherapy (Johnson & Williams, 1980) and we have used such measurements in the PHC xenograft model to assess response to CB3717 treatment. Reliable estimates of tumour volume are difficult to obtain and cannot assess the proportion of necrotic tissue within the tumour, whereas serum AFP is a more sensitive reflection of viable tumour mass. Serial serum AFP measurements of mice bearing the PHC xenografts have shown that high dose CB3717 inhibits the growth of both PLC/PRF/5 and Hep 3B derived xenografts. In addition low dose CB3717 significantly retards the growth of PLC/PRF/5 xenografts. This inhibition of PHC growth by CB3717 may appear less dramatic than the 'cures' obtained in 90% of animals bearing L1210 derived murine tumour (Jones *et al.*, 1981), but it should be emphasised that these workers started CB3717 treatment when the established tumour was about $2 \times 10^6$ cells. By contrast we have allowed the PHC xenografts to grow much larger before starting chemotherapy in order to mimic the situation existing in patients with primary liver cell carcinoma, and have still observed a significant growth inhibitory effect. It is interesting to note that when the very small PLC/PRF/5 derived xenograft was treated with low dose CB3717, complete tumour regression was observed.

The greater sensitivity of PLC/PRF/5 xenografts to CB3717 may be due to a more rapid growth rate. The PLC/PRF/5 xenograft doubling time is ~4.5 days (Figure 4), whereas the Hep 3B xenograft doubling time is ~6.5 days (Figure 6). PLC/PRF/5 xenografts might therefore be expected to have a greater requirement for DNA synthesis and hence de novo TMP synthesis. Alternatively the greater resistance of Hep 3B cells to CB3717 may be due to a greater activity of the salvage pathway in the presence of CB3717. This is supported by the greater increase in thymidine incorporation observed in Hep 3B cells than PLC/PRF/5 cells following CB3717 treatment. In mice there is a high concentration of circulating thymidine (Taylor *et al.*, 1983) so a greater ability of Hep 3B cells to activate thymidine kinase could account for the greater resistance of Hep 3B derived xenografts to CB3717. *In vitro* however the thymidine concentration is much lower, so thymidine kinase activation would not confer such an advantage on Hep 3B cells, rendering the ID50 similar for both PHC cell lines. To test this hypotheses fully thymidine kinase activity, dTTP pool size and thymidine transport in cells needs to be measured. If the salvage pathway is important inhibitors of thymidine transport such as dipyridamole may be of value (Nelson & Drake, 1984). Another possible reason for the greater sensitivity of PLC/PRF/5 cells to CB3717 is that thymidine may be rapidly broken down in these cells by dihydrothymine dehydrogenase. This rate limiting enzyme of thymidine catabolism has been shown to be present in high levels in some rat hepatoma cells (Jackson & Weber, 1976).

The fibrous scarring of the kidneys observed in the mice bearing PHC xenografts is similar to other studies in mice which indicate the dose limiting toxicity is due to CB3717 precipitation in the nephron (Newell *et al.*, 1982). However these preclinical toxicology studies have shown that this can be prevented by alkalinization and clinically it should be possible to avert potential renal toxicity by appropriate scheduling.

The inhibition of growth of two human PLC cell lines both in culture and xenograft models by CB3717 suggests that this quinazoline antifolate

**Figure 6** Effect of CB3717 treatment on growth of Hep 3B derived human liver cell cancer xenografts as measured by (a) relative serum AFP (AFPt/AFPo) and (b) relative tumour volume (Vt/Vo) (●) saline injected controls, (○) 125 mg CB3717 i.p. for 5 days, (△) 200 mg CB3717 i.p. for 5 days. *$P<0.05$.

may be of potential benefit in the chemotherapy of human PHC. A Phase II clinical study in human primary liver cell carcinoma is currently in progress in Newcastle.

This work was supported by the North of England Cancer Research Campaign. We are grateful to ICI for supplying the CB3717, to the Public Health Laboratory Service, Newcastle upon Tyne, for providing facilities in their laboratories, and to Dr H. Calvert for helpful discussion.

### References

ADEN, D.P., FOGEL, A., PLOTKIN, S., DARUJANOV I. & KNOWLES, B.B. (1979). Controlled synthesis of HBsAg in a differentiated human liver carcinoma derived cell-line. *Nature*, **282**, 615.

ALEXANDER, J.J., BEY, E.M., GEDDES, E.W. & LECATSAS, G. (1976). Establishment of a continuously growing cell-line from primary carcinoma of the liver. *S. Afr. Med. J.*, **50**, 2124.

ALISON, D.L., NEWEL, D.R., SESSA, C. & 4 others (1985). The clinical pharmacokinetics of the novel antifolate $N^{10}$-propargyl-5, 8-dideazafolic acid (CB3717). *Cancer Chemother. Pharmacol.*, **14**, 265.

BASSENDINE, M.F., ARBORGH, B.A.M., SHIPTON, U. & 4 others (1980). Hepatitis B surface antigen and alpha-fetoprotein secreting human primary liver cell cancer in athymic mice. *Gastroenterol.*, **79**, 528.

BASSENDINE, M.F., WRIGHT, N.A., THOMAS, H.C. & SHERLOCK, S. (1983). Growth characteristics of alpha-fetoprotein secreting human hepatocellular carcinoma in athymic (nude) mice. *Clin. Sci.*, **64**, 643.

CHABNER, B.A. (1982*a*). Pyrimidine antagonists. In *Pharmacologic Principles of Cancer Treatment*, Chabner, B.A. (ed) p. 183. W.B. Saunders Co., Philadelphia: USA.

CHABNER, B.A. (1982*b*). Methotrexate. In *Pharmacologic Principles of Cancer Treatment*, Chabner, B.A. (ed) p. 229. W.B. Saunders Co., Philadelphia: USA.

CONNICK, T.J., DUNLAP, R.B. & JONES, T.R. (1983). F-NMR investigation of thymidylate synthetase-quinazoline folate interaction. *Fed. Proc.*, **42**, 2107.

CURTIN, N.J. & SNELL, K. (1983). Enzymic retro-differentiation during hepatocarcinogenesis and liver regeneration in rats *in vivo*. *Br. J. Cancer*, **48**, 495.

DANENBERG, P.V. (1977). Thymidylate synthetase – a target enzyme in cancer chemotherapy. *Biochim. Biophys. Acta*, **473**, 73.

DELMONTE, L. & JUKES, T.H. (1962). Folic acid antagonists in cancer chemotherapy. *Pharm. Rev.*, **14**, 91.

DIDDENS, H., NIETHAMMER, D. & JACKSON, R.C. (1983). Patterns of cross resistance to the antifolate drugs trimetrexate, metroprime homofolate and CB3717 in human lymphoma and osteosarcoma cells resistant to metotrexate. *Cancer Res.*, **43**, 5286.

FALKSON, G., MACINTYRE, J.M., MOERTEL, C.G., JOHNSON, L.A. & SCHERMAN, R.C. (1984). Primary liver cancer. An Eastern Cooperative Oncology Group Trial. *Cancer*, **54**, 970.

GORGUN, B. & WATNE, A.L. (1967). Infusion chemotherapy in hepatoma and metastatic liver tumours. *Amer. J. Surg.*, **113**, 363.

HEIDELBERGER, C., CHAUDARI, N.K. & DANNEBERG, P. (1957). Fluorinated pyrimidines: A new class of tumour inhibitory compounds, *Nature*, **179**, 663.

HYRNUIK, W.M. (1975). The mechanism of action of methotrexate in cultured L51787 leukaemia cells. *Cancer Res.*, **35**, 1085.

JACKMAN, A.L., TAYLOR, G.A., CALVERT, A.I.T. & HARRAP, K.R. (1984). Modulation of Anti-metabolite effects: Effects of Thymidine on the efficacy of the quinazoline-based Thymidylate synthetase inhibitor CB3717. *Biochem. Pharmacol.*, **33**, 3269.

JACKSON, R.C., JACKMAN, A.L. & CALVERT, A.H. (1983). Biochemical effects of a quinazoline inhibitor of thymidylate synthetase, N-(4-(N((2-amino-4-hydroxy-6-quinazolinyl)methyl)prop-2-ynylamino)benyozl)-L; glutamic acid (CB3717) on human lymphoblastoid cells. *Biochem. Pharmacol.*, **32**, 3783.

JACKSON, R.C. & WEBER, G. (1976). Enzyme pattern directed chemotherapy: the effects of combinations of methotrexate, 5-fluorodeoxyuridine and thymidine on rat hepatoma cells *in vitro*. *Biochem. Pharmacol.*, **25**, 2613.

JOHNSON, P.J. & WILLIAMS R. (1980). Serum Alpha-fetoprotein estimations and doubling time in hepatocellular carcinoma: influence of therapy and possible value in early detection. *JNCI*, **64**, 1329.

JONES, T.R., CALVERT, A.H., JACKMAN, A.L., BROWN, S.J., JONES, M. & HARRAP, K. (1981). A potent antitumour quinazoline inhibitor of thymidylate syntheatase: synthesis, biological properties and therapeutic results in mice. *Eur. J. Cancer*, **17**, 11.

NAGUSUE, N., YUKAYA, H., HAMADA, T., HIROSE, S., KANASHIMA, R. & INOKUCHI, D. (1984). The natural history of hepatocellular carcinoma. *Cancer*, **54**, 1461.

NELSON, J.A. & DRAKE, S. (1984). Potentiation of methetrexate toxicity by dipyridamole. *Cancer Res.*, **44**, 2493.

NEWELL, D.R., SIDDIK, Z.H., CALVERT, A.H. & 4 others (1982). Pharmacokinetic and toxicology studies with CB3717. *Proc. Am. Assoc. Can. Res.*, **711**, 181.

NIETHAMMER, D. & JACKSON, R.C. (1975). Changes of molecular properties associated with the development of resistance against methotrexate in human lymphoblastoid cells. *Eur. J. Cancer*, **11**, 845.

PROVAN, J.L., STOKES, J.F. & EDWARDS, D. (1968). Hepatic artery infusion chemotherapy in hepatoma. *Br. Med. J.*, **iii**, 346.

TAYLOR, G.A., JACKMAN, A.L., CALVERT, A.H. & HARRAP, K.R. (1983). In *Purine Metabolism in Man IV, Part B. Biochemical, Immunological and Cancer Research*, De Brugn *et al.* (eds) p. 379. Plenum Press: New York.

WATERHOUSE, J., MUIR, C.S., CORREA, P. & POWELL, J. (eds) (1976). Cancer incidence in five countries. *IARC Sci. Publ.*, **3**, 500.

WEBER, G. (1983). Biochemical strategy of cancer cells and the design of chemotherapy. *Cancer Res.*, **43**, 3466.